# A measurement of the hysteresis loop in force-spectroscopy curves using a tuning-fork atomic force microscope

**DOI:** 10.3762/bjnano.3.23

**Published:** 2012-03-08

**Authors:** Manfred Lange, Dennis van Vörden, Rolf Möller

**Affiliations:** 1 Faculty of Physics, University of Duisburg-Essen, Lotharstr.1-21 47048 Duisburg, Germany

**Keywords:** atomic force microscopy, energy dissipation, force spectroscopy, hysteresis loop, PTCDA/Ag/Si(111) √3 × √3

## Abstract

Measurements of the frequency shift versus distance in noncontact atomic force microscopy (NC-AFM) allow measurements of the force gradient between the oscillating tip and a surface (force-spectroscopy measurements). When nonconservative forces act between the tip apex and the surface the oscillation amplitude is damped. The dissipation is caused by bistabilities in the potential energy surface of the tip–sample system, and the process can be understood as a hysteresis of forces between approach and retraction of the tip. In this paper, we present the direct measurement of the whole hysteresis loop in force-spectroscopy curves at 77 K on the PTCDA/Ag/Si(111) √3 × √3 surface by means of a tuning-fork-based NC-AFM with an oscillation amplitude smaller than the distance range of the hysteresis loop. The hysteresis effect is caused by the making and breaking of a bond between PTCDA molecules on the surface and a PTCDA molecule at the tip. The corresponding energy loss was determined to be 0.57 eV by evaluation of the force–distance curves upon approach and retraction. Furthermore, a second dissipation process was identified through the damping of the oscillation while the molecule on the tip is in contact with the surface. This dissipation process occurs mainly during the retraction of the tip. It reaches a maximum value of about 0.22 eV/cycle.

## Introduction

Noncontact atomic force microscopy (NC-AFM) is a powerful tool for the study of surface properties. The invention of the frequency-modulation mode (FM) [1] has made it possible to achieve true atomic resolution [2] with a NC-AFM. In this mode the distance between the sample and the tip is adjusted by maintaining the frequency shift of the cantilever at a constant value while scanning the sample. During operation the oscillation amplitude is kept constant by a second control loop. The amplitude control loop provides valuable information on nonconservative interactions between the tip apex and the sample, which cause damping of the oscillation amplitude [3]. The excitation energy needed to keep the oscillation amplitude constant is directly related to the dissipation.

While the mechanisms of topographic imaging are well understood [4], the dissipation processes on the atomic scale need to be investigated. In general, dissipation can be understood as a hysteresis of forces between approach and retraction of the tip [5–6]. The hysteresis is caused by bistabilities in the potential energy surface of the tip–sample system. Experiments and calculations [7–8] show that dissipation on the atomic level originates from the adhesion or displacement of single atoms caused by strong interaction between the sample and the tip apex.

Simulations for an MgO tip and a MgO surface by L. N. Kantorovich and T. Trevethan [6] showed that the width of such a hysteresis, which may be observed experimentally, depends on the temperature. Due to thermal excitation the width reduces to 1 Å at 100 K and to 0.1 Å at room temperature. The development of NC-AFM instruments that operate at low temperatures and with small amplitudes should enable a direct evaluation of such a hysteresis by analysis of the differences between the force–distance curves during approach and retraction.

In this paper, we report the measurement of hysteresis in force-spectroscopy curves at 77 K with a home-built low-temperature tuning-fork-based AFM (LT-TF-AFM) [9]. When a conductive sample is used, scanning tunneling microscopy (STM) and FM-AFM measurements may be combined. The use of a tuning fork as a sensor allows an oscillation amplitude in the subnanometer regime to be used, due to its large spring constant of about 9000 N/m.

The force-spectroscopy measurements were performed on the organic molecule 3,4,9,10-perylene-tetracarboxylic-dianhydride (PTCDA) grown on a Ag/Si(111) √3 × √3 surface. PTCDA has been extensively studied as a candidate for organic devices [10–15] and its adsorption geometry and binding mechanism is well-known on several surfaces. Furthermore the electronic structure and growth of PTCDA on the Ag/Si(111) √3 × √3 surface is well understood [10,16–17]. In the submonolayer range the PTCDA molecules grow on the Ag/Si(111) √3 × √3 surface in three different phases, namely the herringbone, square and hexagonal phases.

## Results and Discussion

**Figure 1:**
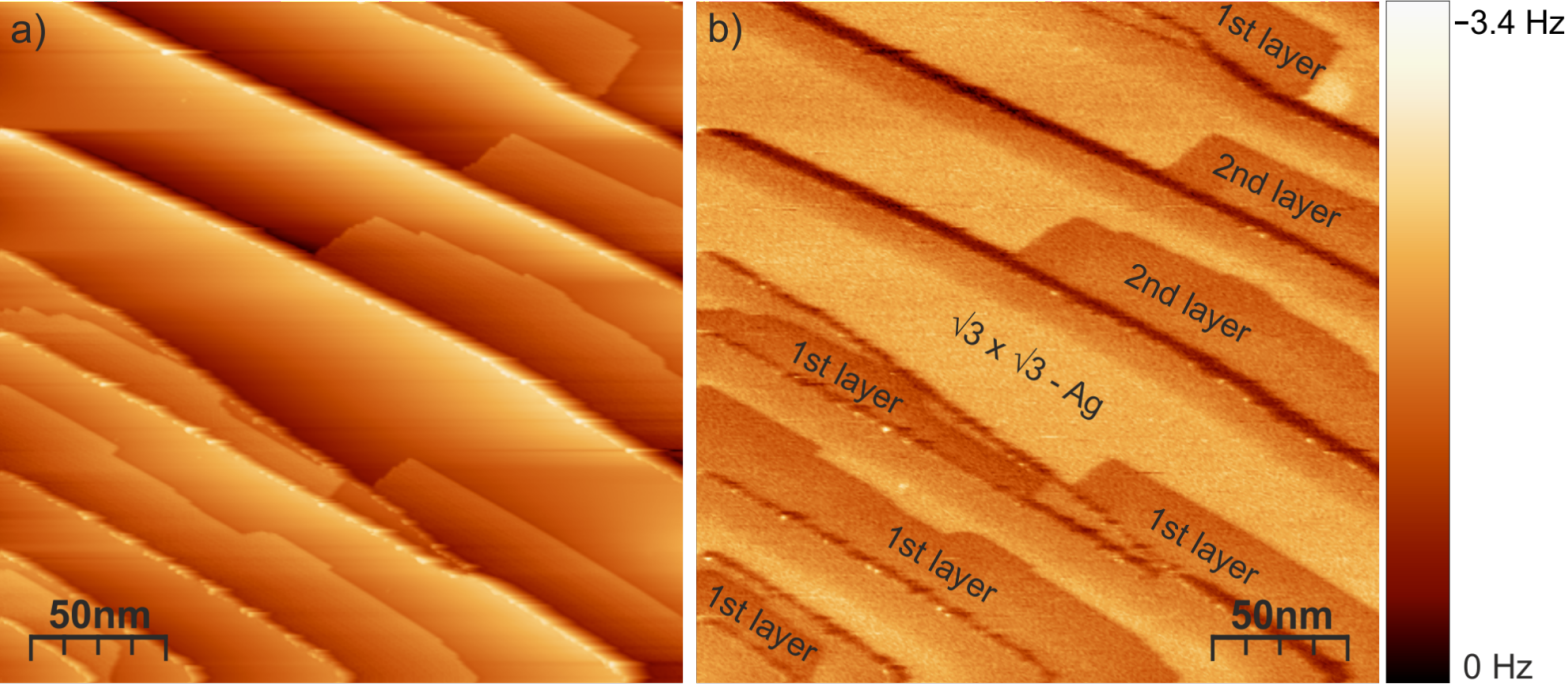
(a) STM image of the PTCDA/Ag/Si(111) √3 × √3 surface. Scan area: 250 nm × 250 nm, tunneling voltage *U* = 0.9 V, tunneling current *I* = 70 pA. (b) Simultaneously recorded frequency shift at an oscillation amplitude of 2.8 Å. The Ag/Si(111) √3 × √3 surface gives rise to a larger frequency shift than the PTCDA islands.

Figure 1a shows an STM 250 nm × 250 nm overview scan of the PTCDA/Ag/Si(111) √3 × √3 surface after the deposition of ~0.3 ML PTCDA. The PTCDA molecules grow from step edges or between steps and form single- or double-layer islands. The PTCDA islands can be clearly distinguished from the Ag/Si(111) √3 × √3 surface by the simultaneously recorded frequency-shift (AFM) image (Figure 1b). The frequency shifts on the PTCDA islands and Ag/Si(111) √3 × √3 surface are about −1 Hz and −2 Hz, respectively. This means that for a given tunneling current the attractive forces are stronger on the Ag/Si(111) √3 × √3 surface than on the PTCDA islands.

**Figure 2:**
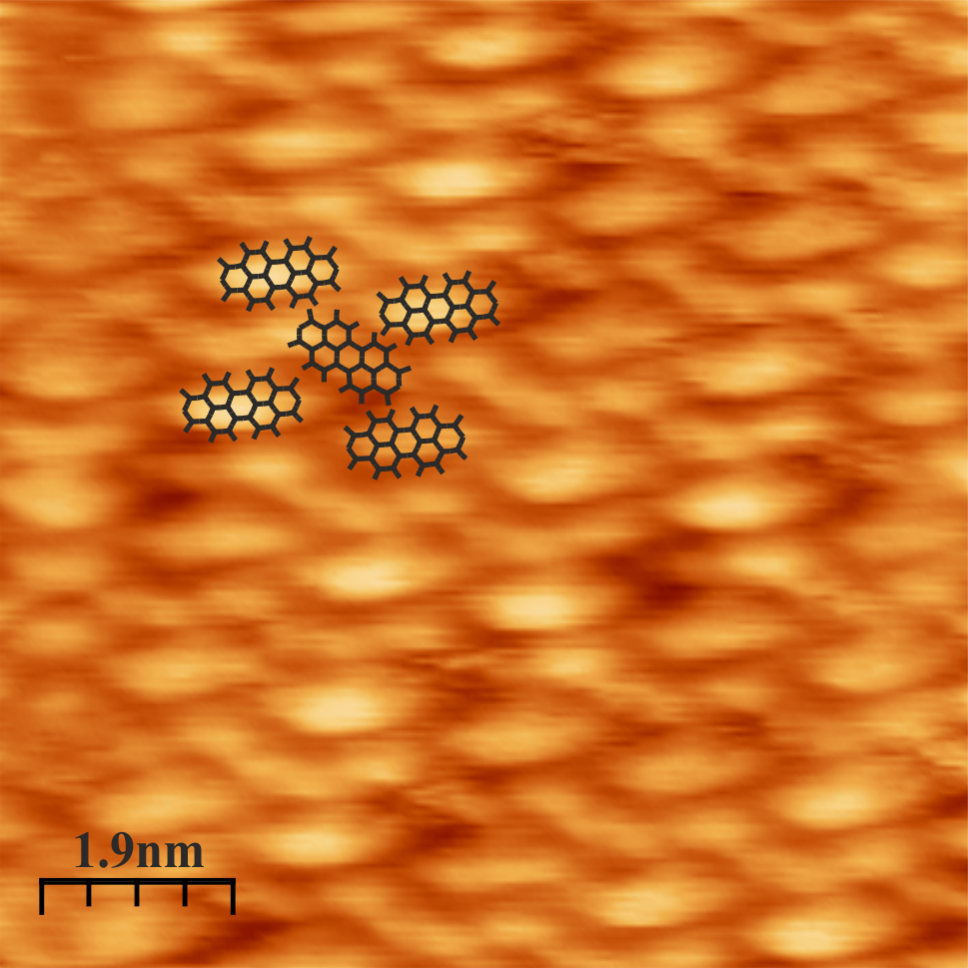
STM image of a double layer of PTCDA arranged in a herringbone phase. The structure is indicated by the schematic drawing of molecular lattice. Scan area: 9 nm × 9 nm; tunneling voltage *U* = 1 V; tunneling current *I* = 60 pA.

Prior to the force-spectroscopy measurements the tungsten tip was prepared by making “soft contact” between the tip and a PTCDA island. Before and after the soft dipping process the area of contact was scanned to ensure that a PTCDA molecule was picked up with the tip apex. The force spectroscopy data presented in this paper are recorded on the double-layer PTCDA island displayed in Figure 2. The PTCDA molecules are arranged in a herringbone phase, as indicated by the drawing of the molecule.

**Figure 3:**
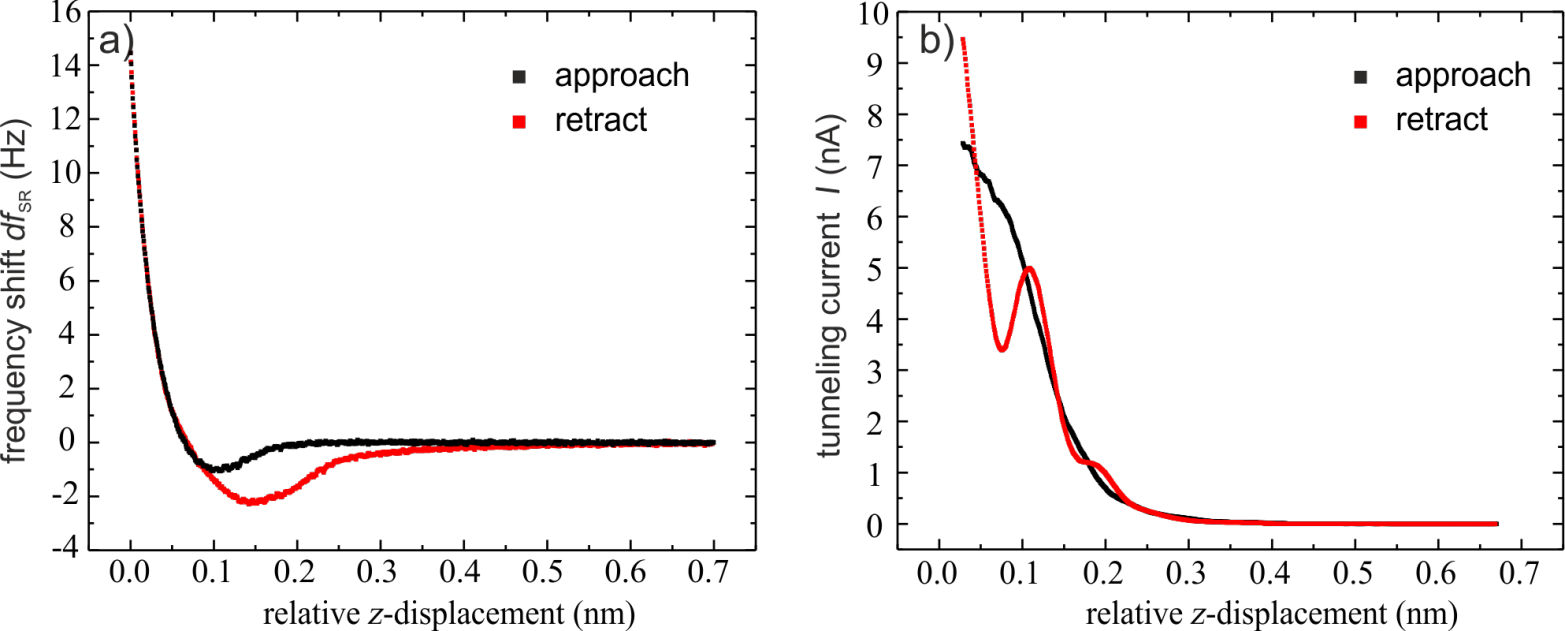
(a) Frequency-shift versus distance curve. The contribution from the long-range forces has been subtracted. The spectroscopy measurement was recorded with an oscillation amplitude of 2.8 Å and a bias voltage of 0.1 V to eliminate the electrostatic interaction. The black curves represent the approach of the tip to the surface, the red curves the retraction. (b) Simultaneously recorded tunneling current.

**Figure 4:**
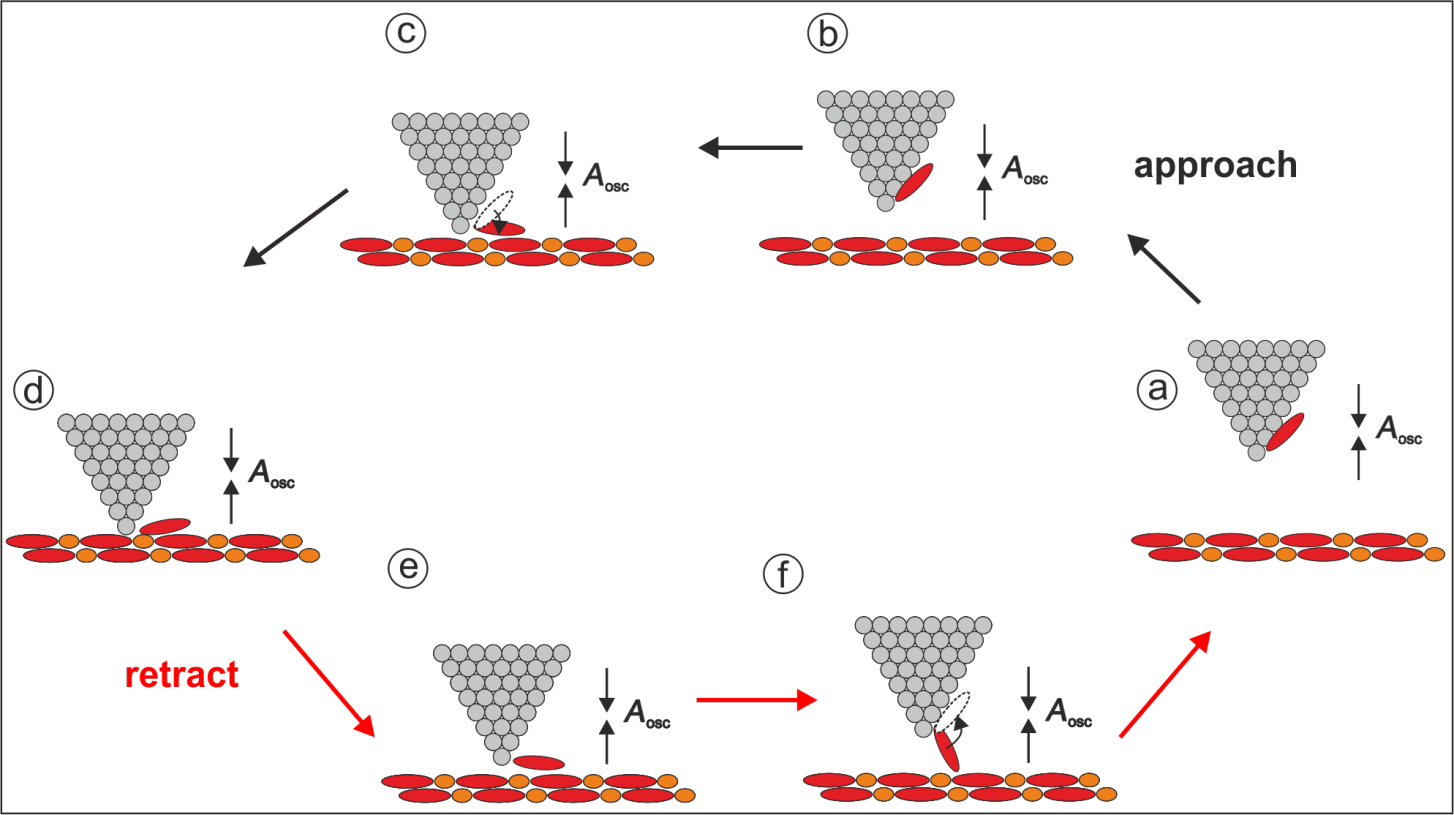
Scheme of the dissipation processes. The black arrows mark the different “snapshots” for the approach of the tip (upper part of the drawing), the red arrows for the retraction (lower part of the drawing). At points (c) and (f) the molecule at the tip abruptly flips to a new position thereby dissipating energy into short-lived vibronic excitations. This energy is given by the work done by the tip as can be seen from the hysteresis in the force distance curves. While the molecule is bridging the gap a second dissipation process occurs, which results in the damping of the oscillation of the tip.

In order to compensate the electrostatic long-range forces, frequency-shift versus bias-voltage curves were recorded on the PTCDA herringbone island, revealing a contact potential difference of 0.1 V. By adjusting the bias voltage to this value the electrostatic long-range forces can be eliminated. The long-range van der Waals (vdW) interaction was determined by fitting the frequency-shift versus distance (*df*–*z*) data at large tip–sample separation by the function (2.8) given in [18]. The vdW fit was extrapolated and subtracted from the *df*–*z* curves, resulting in *df*–*z* curves determined by the pure short-range interaction (*df*_SR_). Figure 3a shows the short-range *df*_SR_–*z* curve measured on the PTCDA herringbone island with an amplitude of 2.8 Å. The black curve represents the approach of the tip towards the surface and the red curve the retraction. The *df*_SR_–*z* curves reveal a hysteresis due to a change of the forces between the tip and sample. To ensure that the hysteresis loop was not induced by a permanent modification of the tip or sample, images of the surface were repeatedly taken before and after each measurement. Since the hysteresis was only observed after the tip had picked up a molecule we assume that it is induced by the PTCDA molecule on the tip apex. Most likely the molecule on the tip apex forms a bond with one or more molecules of the herringbone island when the tip comes very close to the surface. This bond is successively broken when the tip is retracted. The width of the hysteresis loop is about 3–4 Å, hence larger than the oscillation amplitude of the tuning fork. The width corresponds to a geometric change in the tip–sample configuration, which is probably caused by the lifting of the molecule. A schematic representation of the process is displayed in Figure 4.

The simultaneously measured tunneling current is shown in Figure 3b. It was calculated from the recorded time-averaged tunneling data by using the script of J. E. Sader and Y. Sugimoto [19]. Since it does not show a significant hysteresis, it may be concluded that the main contribution to the current does not flow through the molecule. Although there are minor kinks in the curve of the tunneling current when the tip approaches the surface, these cannot be uniquely attributed to bond formation by the PTCDA molecule. When the tip was retracted from the surface a significant dip in the tunneling current was found. By comparison to other measurements this may be attributed to the molecule between the tip and the surface.

**Figure 5:**
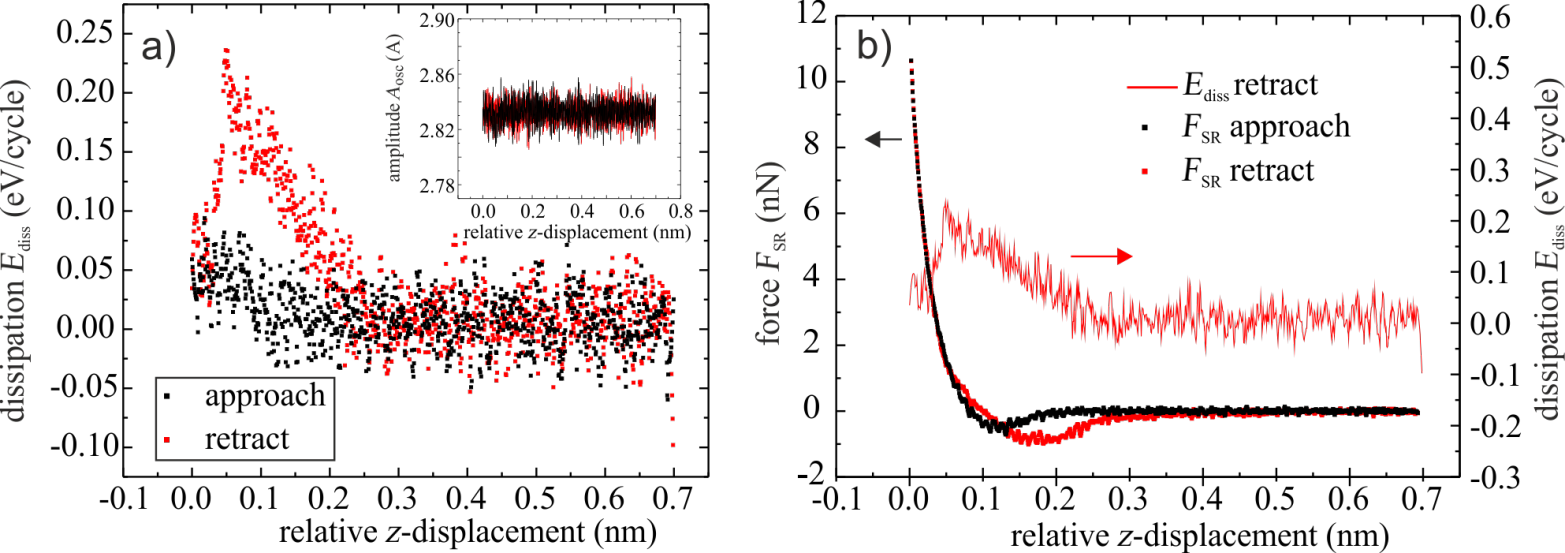
(a) Dissipation signal for approach (black dots) and retraction (red dots). The inset displays the simultaneously recorded oscillation amplitude. The maximum in the dissipation signal is found during the retraction. (b) Dissipation signal for the retraction together with the calculated force–distance curves.

A second dissipation process is observed through the damping of the oscillation of the tip. It occurs in every cycle of the oscillation, hence many thousands of times during the measurement of the force–distance curves. Figure 5a shows the dissipation signal (*E*_diss_) for the approach (black dots) and the retraction (red dots) measured as the power needed to maintain a constant amplitude. As can be seen from the inset, the simultaneously measured oscillation amplitude varies by less than +/− 1%. During the approach of the tip this type of dissipation starts very close to the surface and becomes more prominent upon retraction, reaching a maximum value of 0.22 eV/cycle. It decreases with increasing distance and vanishes at a distance of 0.25 nm from the closest approach. Figure 5b displays the dissipation signal of the retraction together with the force–distance (*F*_SR_–*z*) spectrum calculated from the *df*_SR_–*z* data of Figure 3a by using the script by J. E. Sader and S. P. Jarvis [20]. The damping of the oscillation is observed within the distance range that corresponds to the hysteresis in the force–distance curves, i.e., where the forces for approach and retraction of the tip are different. As sketched in Figure 4, it is rather intuitive that this dissipation is associated with the motion of the molecule between the tip and the sample. It is interesting to compare the energy for this process to the energy associated with the making and breaking of the bond between the PTCDA molecule and the surface, which is given by the area of the hysteresis loop [5–6]. The dissipated energy of this process was determined to be approximately 0.57 eV, which is of the order of the energy of a chemical bond. In [21] the value of about 1 eV is given for the lifting off of the PTCDA molecule completely from the surface. Hence, the observed energy fits well for breaking the bond of a molecule that is partially bound to the surface. It is about two times larger than the maximal energy dissipated by the molecule during the oscillation of the tip.

Figure 4 illustrates a model to explain the two dissipation processes during the approach and retraction of the tip. The black arrows (upper path of the drawing) represent the approach corresponding to the black curve in Figure 5b. The red arrows in the drawing indicate the retraction and correspond to the red curves in Figure 5b. The bond formation between the PTCDA molecule at the tip and the molecules of the surface occurs in Figure 4c. Once the molecule is situated between the tip and the surface the oscillation of the tip is damped. During further approach of the tip towards the surface this dissipation increases. During the retraction of the tip the dissipation reaches a maximum before the bond is finally broken at much larger distance (Figure 4f). At that point the damping of the oscillation vanishes and there is no significant difference in the forces between the forward and backward directions. In contrast to the breaking of the bond of an individual atom, the binding of the molecule to the surface results from the superposition of many contributions and this binding can be successively torn apart, which may explain why no abrupt change is observed in the force–distance curve.

## Conclusion

We have resolved the hysteresis loop in force-spectroscopy measurements induced by the bond formation and breakage between a PTCDA molecule at the tip of an NC-AFM probe and PTCDA molecules of the sample surface. The dissipated energy of this process is given by the area of the hysteresis loop and was determined to be 0.57 eV. While the molecule is situated between the tip and the surface the oscillation of the tip of the NC-AFM is damped. The dissipation energy of this process is 0.22 eV/cycle at maximum.

## Experimental

The experiments were performed at 77 K under ultrahigh vacuum (UHV) conditions. Measurements were performed using a home-built LT-TF-AFM [9], which is able to operate both as an STM and as an FM-AFM. The tuning fork is used in the qPlus configuration [22]. The oscillation amplitude of the tuning fork can be chosen in the subnanometer regime due to its large spring constant of about 9000 N/m, preventing a jump to contact. This offers the advantage that in this regime the measurements are more sensitive to short-range forces and dissipation processes. The resonance frequency and quality factor of the tuning fork at 77 K temperature and under UHV conditions are about 28 kHz and 10000, respectively.

The tuning fork and tunneling signal are wired separately to avoid any crosstalk. Both signals are amplified by home-built current-to-voltage converters outside the vacuum system. For the detection and regulation of the tuning-fork oscillation a phase-locked-loop system supplied by Specs Zürich (Nanonis) is used. Scanning control and data acquisition are performed by the open-source software GXSM [23] combined with home-built electronics. For image processing the free software WSXM [24] is used.

Sample preparation was performed as follows. Si(111) was flash annealed at 1500 K by direct resistive heating. The sample was then cooled down slowly from 1200 K to 800 K to prepare the 7 × 7 reconstruction. Next, 1.0 ML of Ag was evaporated while the Si reconstruction was kept at 800 K. This results in a surface covered by the √3 × √3 reconstruction [25]. Finally, ~0.3 ML PTCDA was deposited while the sample was at room temperature.

## References

[1] Albrecht T R, Grütter P, Horne D, Rugar D (1991). J Appl Phys.

[2] Giessibl F J (1995). Science.

[3] Mortia S, Wiesendanger R, Meyer E (2002). Noncontact Atomic Force Microscopy.

[4] Giessibl F J (1997). Phys Rev B.

[5] Sasaki N, Tsukada M (2000). Jpn J Appl Phys, Part 2.

[6] Kantorovich L N, Trevethan T (2004). Phys Rev Lett.

[7] Oyabu N, Pou P, Sugimoto Y, Jelinek P, Abe M, Morita S, Pérez R, Custance Ó (2006). Phys Rev Lett.

[8] Li Y J, Nomura H, Ozaki N, Naitoh Y, Kageshima M, Sugawara Y, Hobbs C, Kantorovich L (2006). Phys Rev Lett.

[9] Wintjes N, Lange M, van Vörden D, Karacuban H, Utzat D, Möller R (2010). J Vac Sci Technol, B: Microelectron Nanometer Struct–Process, Meas, Phenom.

[10] Swarbrick J C, Ma J, Theobald J A, Oxtoby N S, O’Shea J N, Champness N R, Beton P H (2005). J Phys Chem B.

[11] Fendrich M, Kunstmann T (2007). Appl Phys Lett.

[12] Wagner T, Bannani A, Bobisch C, Karacuban H, Möller R (2007). J Phys: Condens Matter.

[13] Wagner T, Bannani A, Bobisch C, Karacuban H, Stöhr M, Gabriel M, Möller R (2004). Org Electron.

[14] Kilian L, Hauschild A, Temirov R, Soubatch S, Schöll A, Bendounan A, Reinert F, Lee T-L, Tautz F S, Sokolowski M (2008). Phys Rev Lett.

[15] Kunstmann T, Schlarb A, Fendrich M, Wagner T, Möller R, Hoffmann R (2005). Phys Rev B.

[16] Gustafsson J B, Zhang H M, Johansson L S O (2007). Phys Rev B.

[17] Zhang H M, Gustafsson J B, Johansson L S O (2010). Chem Phys Lett.

[18] Guggisberg M, Bammerlin M, Loppacher C, Pfeiffer O, Abdurixit A, Barwich V, Bennewitz R, Baratoff A, Meyer E, Güntherodt H-J (2000). Phys Rev B.

[19] Sader J E, Sugimoto Y (2010). Appl Phys Lett.

[20] Sader J E, Jarvis S P (2004). Appl Phys Lett.

[21] Fournier N, Wagner C, Weiss C, Temirov R, Tautz F S (2011). Phys Rev B.

[22] Giessibl F J (1998). Appl Phys Lett.

[23] Zahl P, Bierkandt M, Schröder S, Klust A (2003). Rev Sci Instrum.

[24] Horcas I, Fernández R, Gómez-Rodríguez J M, Colchero J, Gómez-Herrero J, Baro A M (2007). Rev Sci Instrum.

[25] Wan K J, Lin X F, Nogami J (1993). Phys Rev B.

